# Effects of Adaptogens on the Central Nervous System and the Molecular Mechanisms Associated with Their Stress—Protective Activity

**DOI:** 10.3390/ph3010188

**Published:** 2010-01-19

**Authors:** Alexander Panossian, Georg Wikman

**Affiliations:** Swedish Herbal Institute Research & Development, Spårvägen 2, SE-432 96 Åskloster, Sweden

**Keywords:** adaptogens, herbal medicine, fatigue, Hsp70, neuroprotection, clinical trials

## Abstract

Adaptogens were initially defined as substances that enhance the “state of non-specific resistance” in stress, a physiological condition that is linked with various disorders of the neuroendocrine-immune system. Studies on animals and isolated neuronal cells have revealed that adaptogens exhibit neuroprotective, anti-fatigue, antidepressive, anxiolytic, nootropic and CNS stimulating activity. In addition, a number of clinical trials demonstrate that adaptogens exert an anti-fatigue effect that increases mental work capacity against a background of stress and fatigue, particularly in tolerance to mental exhaustion and enhanced attention. Indeed, recent pharmacological studies of a number of adaptogens have provided a rationale for these effects also at the molecular level. It was discovered that the stress—protective activity of adaptogens was associated with regulation of homeostasis via several mechanisms of action, which was linked with the hypothalamic-pituitary-adrenal axis and the regulation of key mediators of stress response, such as molecular chaperons (e.g., HSP70), stress-activated c-Jun N-terminal protein kinase 1 (JNK1), Forkhead box O (FOXO) transcription factor DAF-16, cortisol and nitric oxide.

## 1. Introduction

**Figure 1:**
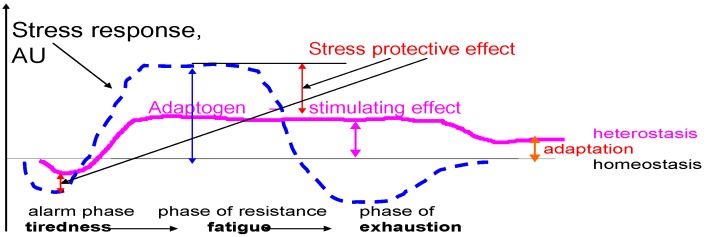
Adaptogens increase the state of non-specific resistance in stress and decrease sensitivity to stressors, which results in stress protection, and prolong the phase of resistance (stimulatory effect). Instead of exhaustion, a higher level of equilibrium (the homeostasis) is attained the heterostasis. The higher it is, the better the adaptation to stress. Thus, the stimulating and anti-fatigue effect of adaptogens has been documented in both in animals and in humans. Adapted from [26,78,133].

The idea that a pill could improve mental and physical performance in healthy people was devised during World War II with various stimulants given to pilots and members of submarine crews [1,2,3,4,5,6]. For instance, the first studies on the stimulating and tonic effects of *Schisandra* *chinensis* were published in Soviet Union WWII military journals [5,6]. The Russian interest in *S.* *chinensis* arises from ethnopharmacological investigations by Komarov (1895) and Arsenyev (1903–1907) in the Far East regions. It was discovered that the berries and seeds were used by Nanai (Goldes or Samagir) hunters as a tonic, to reduce thirst, hunger and exhaustion, and to improve night vision [7,8,9,10]. During the period 1950–60, the idea of using herbal medicinal plants to increase stamina and survival in harmful environment was developed, and a new concept of “adaptogens” was introduced by the toxicologist Lazarev to describe compounds which could increase “the state of non-specific resistance” in stress [11,12]. This concept was based on Hans Selye’s theory of stress and general adaptation syndrome, which have three phases: alarm phase, phase of resistance and phase of exhaustion [13], Figure 1.

At the end of the 1960s Brekhman and Dardimov proposed that adaptogens are innocuous agents, nonspecifically increasing resistance against physically, chemically, biologically and psychologically noxious factors (“stressors”), normalizing effect independent of the nature of pathologic state [14]. In the early 1960’s, the study of adaptogens developed into a field of biomedicinal research in its own right in the USSR. This was due to a major targeted project or direction of research, such as mapping or screening of biologically active substances from the plant kingdom. The aim of the stress research was to develop drugs and methods able to stimulate the intrinsic adaptive mechanisms of the organism to help it survive in situations of intense or prolonged stress, whilst preferably maintaining the capability for physical and mental work [15]. The extent of the research carried out was enormous with 1009 studies (primarily pharmacological and clinical) published in Russia up until 1982 and most of them concerned extracts or isolates prepared from *Eleutherococcus* *senticosus* [16].

Today, research into adaptogens comprises the following four areas: (a) phytochemistry: isolation and structure elucidation of active constituents of adaptogenic plants; (b) biochemistry and molecular biology: mechanisms of stress protective activity of adaptogens on the molecular and cellular levels; (c) experimental and clinical pharmacology: efficacy and safety of adaptogens in stress related disorders on animals and humans; (d) pharmaceutical development of herbal preparations/products that have well established medicinal use in evidence based medicine. Some of the most interesting developments are studies that clearly indicate that certain adaptogenic substances can activate the protective mechanisms of cells, which is linked to an increase in survival rate both *in* *vitro* and *in* *vivo* [17,18,19,20]. These studies have so far been directed at the regulation of molecular chaperons (Heat Shock Proteins), such as Hsp70 and other key stress mediators [21,22,23].

As a pharmacotherapeutic group adaptogens were recently defined as herbal preparations that increased attention and endurance in fatigue, and reduced stress-induced impairments and disorders related to the neuro-endocrine and immune systems [23,24]. This definition was based on evidence obtained from clinical trials, which we evaluated in accordance with the European Medicines Agency Assessment Scale [25] and US Natural Standards Evidence–based Validated Grading Rationale [http://www.naturalstandard.com/grading.html].

The term adaptogen is often applied to plants, even when the criteria of an adaptogen is not met with, such as the important and significant general adaptive effect on stress involving the whole organism and its main organ and functions. In this review article, we will focus our attention on some of the most extensively studied adaptogens: *Rhodiola* *rosea,* *Schisandra* *chinensis* and *Eleutherococcus* *senticosus* [26,27,28,29,30,31,32,33,34,35,36,37,38] and discuss the results of pharmacological and clinical studies, which are relevant to the main topic of discussion.

The normal paradigm one drug for one disease is not appropriate for adaptogens as they can have numerous pharmacological effects and indications. Table 1 and Table 2 show their pharmacological profiles, which are different but similar in terms of their stress-protective action. Therefore, all these pharmacological effects can be combined into the groups associated with stimulating and stress-protective effects in CNS and vegetative nervous systems, endocrine system and immune system, comprising by definition the parts of neuroendocrine-immune complex - stress-system, Table 3 [26].

The adaptogens *Eleutherococcus* *senticosus*, *Rhodiola* *rosea* and *Schisandra* *chinensis* were reported to be safe in acute and subacute toxicity studies. Moreover, adaptogen induced state of non-specific resistance to highly toxic chemicals (e.g., chlorophos, phosphorus, cyclophosphane, strychnine, aniline, sodium nitrite, narcotics like sodium barbital, hexenal, chloralhydrate, benzene, acetone, ether, etc.) and microbes demonstrated in many pharmacological/toxicological studies [28], actually implies that they have an anti-toxic activity. For example, it was demonstrated that repeated administration of *Rhodiola* *rosea* extract during 10 consecutive days decreased LD50 of 40% ethanol in mice from 24.1 ml/kg to 55.2 ml/kg. It was also shown that salidroside shortened (from 100% to 19%) the duration of benzene induced sleep in rats [28].

**Table 1:** Pharmacological profile of adaptogens: summary of *in* *vitro* or in animal studies.

Regulatory System: effect	Pharmacological Effects	*Rhodiola*	*Eleutherococcus*	*Schisandra*
Stress-system (neuro-endocrine-immune complex): Anti-stress/stress-mimetic/ stress-protective	CNS-stimulating: enhancing of physical performance, cognitive performance (learning and memory)	+	+	+
	Neuroprotective	+		+
	Hepatoprotective	+	+	+
	Cardioprotective	+		+
	Gastroprotective		+	+
	Oxidative stress/Radioprotective	+	+	+
	Anti-atherosclerosis		+	+
	Vasodilatatory/hypotensive			+
	Anti-hyperglycemic		+	
	Anti-inflammatory/allergy	+	+	+
	Immunotropic	+	+	+
	Antidepressive	+		
	Anxiolitic	+	+	

**Table 2:** A selected pharmacological profile of adaptogens, clinical efficacy in humans relative to CNS.

	Pathophysiological condition	*Rhodiola*	*Eleutherococcus*	*Schisandra*
Neuro-endocrine system	Physical fatigue	+	+	++
	Mental fatigue (declined attention)	++	+	+
	Stress induced chronic fatigue	+	+	
	Depression	+		

**Table 3:** Summary of the pharmacological activities of *Schisandra* *chinensis,* adapted from [26].

Regulatory system		Pharmacological effect	
Stress-system	Central and vegetative nervous system	Stimulating effect	Adapto-genic effect
	Endocrine system	Stress-mimetic and stress-protective effect	
	Immune system	Stress protective effect	

## 2. Active Compounds

**Figure 2:**
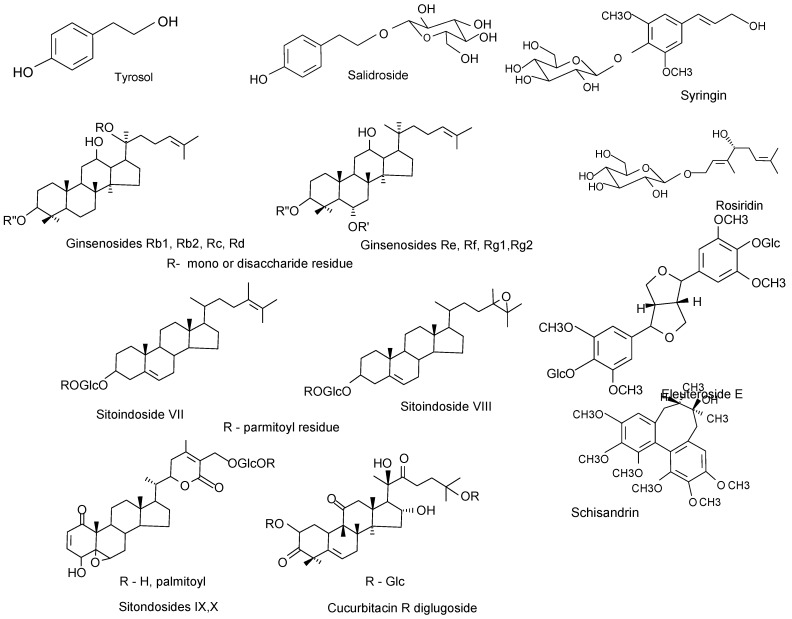
Active compounds isolated from *Rhodiola* *rosea* (tyrosol, salidroside, rosiridin), *Eleutherococcus* *senticosus* (eyringin, eleutheroside E), *Schisandra* *chinensis* (schizandrin), Panax ginseng (ginsenosides), *Withania* *somiphera* (sitoindosides), *Bryonia* *alba* (cucurbitacin R glucoside).

Chemically, the adaptogens are typically either complex phenolics or tetracyclic triterpenoids/ steroids (Figure 2). The phenolic compounds include phenylpropanoids and phenylethane derivatives, such as salidroside (rhodioloside), rosavin, syringin, triandrin, tyrosol, [28,35,38,39,40,41,42,43] and lignans, such as eleutherosid E [44,45,46,47,48] and schisandrin B [49,50,51,52,53,54,55,56,57,58,59,60]. They are structurally similar to the catecholamines -the mediators of the sympathoadrenal system (SAS) involved in activation of the stress system in the early stages of stress response. The tetracyclic triterpenoids, such as cucurbitacin R diglucoside [61,62], ginsenosides [63,64,65,66,67,68] and phytosterol-glycosides (e.g, SG, eleutheroside A, sitoindosides, daucosterol) [27,69,70,71] structurally resemble the corticosteroids that act as stress hormones involved in protective inactivation of the stress system [72,73,74]. In addition, the monoterpene glucoside rosiridin, which is isolated from *Rhodiola* *rosea*, was found to inhibit monoamine oxidases A and B *in* *vitro* implying its potential beneficial effect in depression and senile dementia [75].

Salidroside - the active principles of *R.* *rosea* extracts - was found to have neuro-protective activity, which reduced stress induced impairments and disorders related to the neuro-endocrine and immune system through:

- Stimulation of CNS system [28,39,76,77,78].
- Protection of cultured neuronal cells from sodium azide and glutamate induced injuries [79,80].
- Attenuation of glutamate-induced apoptotic cell death in primary cultured hippocampal neurons of rats [81].
- Blockage of H_2_O_2_**-**induced apoptosis in rat neuronal PCl2 cells [82].
- The effect of anti-neuronal apoptosis related to its function of decreasing intracellular free calcium concentration [83,84].
- Protection of rat neuronal PCl2 cells against amyloid beta-peptide (A beta)-induced cytotoxicity reduced accumulation of reactive oxygen species and malondialdehyde (MDA) [85].
- Stimulation of glucose uptake in skeletal muscle cells by activating phosphorylation of AMP-activated protein kinase [86].
- Protection from oxidative damage during fatigue [87].
- Reduction of the degree of cerebral edema in rats with global cerebral ischemia-reperfusion injury, relieves the metabolism abnormity of free radical and improves the function of cognition [88].

A number of these findings might raise the possibility of potential therapeutic applications of salidroside in preventing and treating cerebral ischemic and neurodegenerative diseases [89].

Tyrosol - another active principle of *Rhodiola* extract - increases phosphorylation of eNOS and FOXO3a, which are key molecular targets involved in this mechanism. Furthermore, it has recently been shown that tyrosol induces the expression of the longevity protein SIRT1 [90].

It is noteworthy that administration of the amino acid tyrosine, which is a common precursor of biosynthesis of tyrosol, salidroside and catecholamines (Figure 2), alleviates both stress-induced depletion of brain catecholamines (norepynephrine and dopamine in the alarm phase of stress syndrome) and reduces fatigue, as noted in animal task performances [91]. A number of clinical studies suggest that supplementation of tyrosine might improve stress-induced (e.g*.*, cold, noise, anxiety and fatigue) accuracy of mental performance [92].

Indeed, schisandrin B has a similar pharmacological profile associated with stress-protective activity. Apparently the neuroprotective effect of schisandrin B [54,55,59,93,94] is associated with the expression of heat shock proteins Hsp70. It has been demonstrated that schisandrin B stimulate the expression of Hsp70 in normal cells [95,96,97,98,99,100,101], which is associated with the enhancement of mitochondrial glutathione status [95,97,100,101,102,103], antioxidant activity [59,96,104,105,106,107,108,109], ATP generation [110], mitigation of age-related impairments in mitochondrial antioxidant status and functional ability in various tissues, enhancement in cognitive functions and an increase in the survival of aging in rodents [56,97,112].

## 3. Antistress and Stimulating Activity of Adaptogens in Animal Model Systems

A typical pharmacological assessment of adaptogens includes the evaluation of stimulating, tonic and stress-protective activities in model animal systems, which were subjected to various stress conditions [112,113].

### 3.1. Anti-Stress Effect of Adaptogens

The stress protective effect of adaptogens has been demonstrated on simple organisms and on isolated cells [17,18,114,115]. Thus, there may be an association with regulation and homeostasis of the neuro-endocrine-immune complex. In addition, there may also be a connection with more evolutionary older congential mechanisms of regulation in cellular homeostasis and the adaptive/defense response to external stressors. We hipothezise that this type of defence system is apparently common for all cells and living organisms and probably includes heat shock proteins among the number of key mediators of innate nonspecific resistance to stressors.

**Figure 3:**
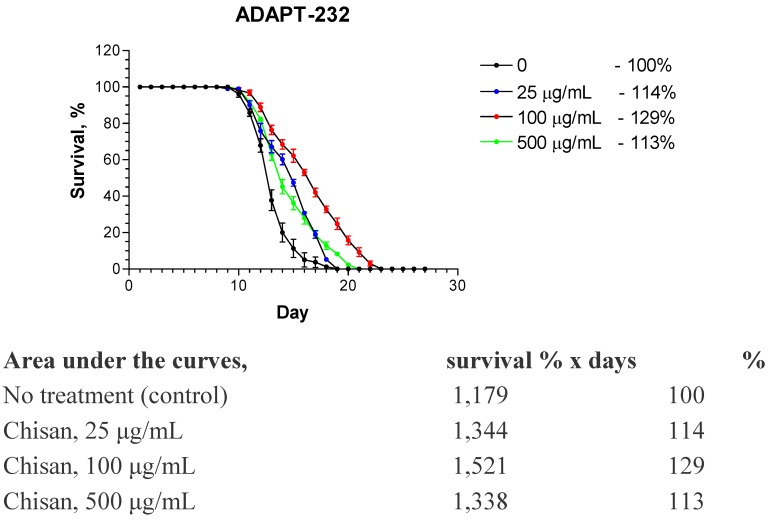
Fixed combination of *Rhodiola* *rosea*, *Schisandra* *chinensis* and *Eleutherococcus* *senticosus* extracts (ADAPT-232/Chisan) causes a concentration-dependant increase in life span of N2 wild-type *C.* *elegans*. A highly significant change in longevity is observed when Kaplan-Meier survival analysis (log-rank test) is used to compare between groups (p = 0.001) (Panosyan and Wiegant, unpublished data, 2005).

It has been shown that the same mechanism can be found in stress tolerance and lifespan extension, which makes them parallel phenomenon Therefore, it is not surprising that adaptogens prolong the lifespan of the nematode *Caenorhabditis* *elegans* [18] and *Drosophila* *melanogaster* in a dose dependent manner [19], e.g., see Figure 3, where effect of ADAPT-232—a fixed combination of *Rhodiola* *rosea*, *Schisandra* *chinensis* and *Eleutherococcus* *senticosus* extracts—on the life splan of *C.elegance* is demonstrated.

### 3.2. Molecular Mechanisms behind Anti-Stress Effect of Adaptogens

**Figure 4:**
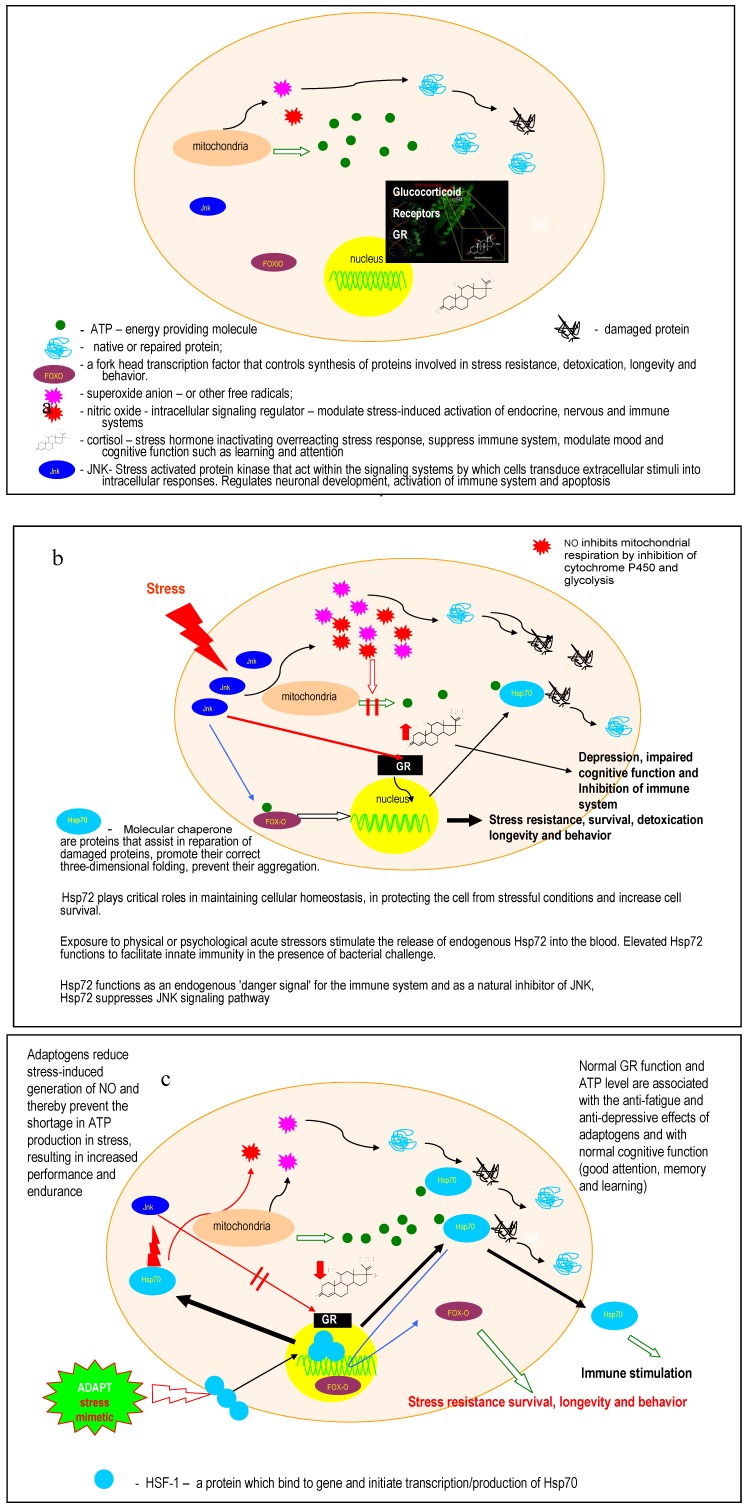
A simplified schematic showing the hypothetical molecular mechanism of ADAPT-232 as it induces stress resistance (adaptation to stress) and enhances cognitive functions and, possibly, longevity. Adapted from [23].

It has been demonstrated that the beneficial stress-protective activity of adaptogens was associated with the hypothalamic-pituitary-adrenal axis and the regulation of key mediators of the stress response common to all cells, of such as:

- Heat shock proteins Hsp70 and Hsp16 [17,22,23,95,96,97,98,99,100,101,116,117,118,119,120], which are molecular chaperons involved in stress-induced cytoprotection and in adaptation of repeated exposure to an initial stressor.
- Stress- activated c-Jun *N*-terminal protein kinase 1 (JNK1) [21,121].
- Forkhead box O (FOXO) transcription factor DAF-16 [17,90].
- HPA- axis, including cortisol and glucocorticoid receptors [21,123,124].
- Beta-endorphin [125,126,127].
- Nitric oxide [21,128,129,130,131].
- The biosynthesis of ATP, thus inducing an alteration in energy source [23,131], Figure 4.

Typically, a cell is either in:

- balance (dynamic equilibrium - homeostasis), or
- functioning under stressful conditions (threatened homeostasis-imbalance),.
- the state of adaptation (tolerance) to stress (*i.e.*, state of non-specific resistance to stress; heterostasis or homeostasis with a higher level of equilibrium),
- the state of apoptosis (dying).

Panel (a) shows that mitochondria generate aggressive oxygen-containing radicals that can damage native or repair proteins by distorting their 3-D structure, so that they can no longer fulfil their functions in the cell.

There are many “players’ involved in the regulation of homeostasis at both the cellular level and the organism level, such as:

- the stress hormone cortisol (a molecule that is secreted from glands and regulates the functions of organs and systems of the organism).
- glucocorticoid receptors that modulate/regulate cortisol secretion (feedback regulation).
- NO, an intracellular signalling molecule that mediates stress response and modulates stress-induced activation of hormonal, nervous and immune systems.
- FoxO, a Forkhead protein that controls the synthesis of proteins involved in stress resistance, cell survival and longevity.

Panel (b) shows that under stress (e.g., infection, cold, heat, radiation, physical load, emotional stress) an external stress signal activates a cascade of “signalling” proteins/enzymes including JNK, a stress-activated enzyme that plays important roles in the regulation of a diverse array of cellular functions such as neuronal development, activation of the immune system and programmed cell death (apoptosis). The functions of JNK are as follows:

- To increase the formation of aggressive radicals and nitric oxide, which in turn suppresses the generation of energy providing molecules (ATP). As a result of lack of energy, many proteins cannot work, several factions are suppressed, and the first symptoms of fatigue and exhaustion are observed. ATP is also required for the normal functioning of heat shock proteins (e.g., Hsp70), which are produced as a defence response to stress and assist in the repair of misfolded and damaged proteins.
- To suppress glucocorticoid receptors (GR) such that the feedback inhibition of cortisol secretion ceases to function and levels of circulatory cortisol increase. The cortisol inhibits the immune system, and has anti-inflammatory effects on the body. It is also required to protect the organism from over-reaction/over-activation in response to stress. However, chronically high levels of cortisol are associated with depression, chronic fatigue and impaired cognitive function, such as decreased attention and learning ability.
- To activate translocation of FoxO to the nucleus and initiates the synthesises of proteins that confer stress-resistance and increased longevity.

Panel (c) shows that adaptogens such as ADAPT-232 decrease NO, cortisol and JNK under stress and stimulate/activate the expression of Hsp70 and p-FoxO1. The stimulation of Hsp70 biosynthesis is a key point in the mechanism of action of adaptogens since the heat shock protein:

- enhances the repair of damaged proteins.
- inhibits the stress-induced expression of NO genes. ATP is increased to normal levels in the adapted cell. This is due to the inability of reduced levels of NO to suppress the formation of energy providing molecules.
- inhibits JNK and consequently apoptotic death and suppression of immune system via activation of GR and other mechanisms. Normal GR function and normal ATP levels are associated with the anti-fatigue and anti-depressive effects of adaptogens and with normal cognitive function (e.g., good attention, memory and learning).
- is probably associated with the effect of adaptogens on the phosphorylation of FoxO and its translocation into the nucleus of isolated cells (*i.e.,* human monocytes) or simple organisms (*i.e.,* DAF-16 in *C.* *elegance*) and, consequently, with increased resistance to stress and increased life span.

In summary, ADAPT-232 works like a stress vaccine (stress-mimetic) by activating stress-induced self-defence mechanisms in order to adapt the cell and organism to mitigate stress-induced harmful effects.

It seems that activation of Hsp70 expression is a key point in the mode of action of adaptogens [23]. We have demonstrated that adaptogens induce an increase of serum Hsp72 in animal studies [22]. This induction is considered as a defense response to stress, which increases tolerance to stress in a combination of physical and emotional stresses. Our data suggest that increased tolerance to adaptogen-induced stress is associated with its stimulation of expression of circulating serum Hsp72 [22]. In fact, Hsp72 production and release is a known mediator of the stress response involved in repairing proteins during physical load. Our working hypothesis is that adaptogens adapt (or make less sensitive) the organism to stress. Thus, adaptogens act like low molecular weight “vaccines” or stress-mimetics, which induce mild activation of the stress system in order to cope with more severe stress. The adaptogens act as challengers and mild stressors (stress-mimetics) [23]. This gives rise to adaptive and stress-protective effects, which are mainly associated with the HPA axis, a part of the stress system that also contributes to the nervous, cardiovascular, immune, gastrointestinal and endocrine systems [64,132,133].

The anti-depressive effect of *Rhodiola* *rosea* [134] may be associated with parts of the stress-system (e.g., secretion of cortisol and the JNK mediated effects on the glucocorticoid receptors) [21,122,134] through its effect on mono-amine oxidase A [75].

A recent review [135] focused on the neuroprotective effects of adaptogens. The authors concluded that the adaptogens *Eleutherococcus* *senticosus*, *Rhodiola* *rosea* and *Schisandra* *chinensis* were all involved in the protection of brain neurons from various injuries, which means that they may have an influence on neurodegenerative mechanisms in Parkinson’s disease [135].

### 3.3. CNS Stimulatory Effect

Apparently, stimulating (acute/single dose effect) and tonic (effect of repeated/multiple administration) effects of adaptogens are actually consequences of their stress-protective activity. CNS stimulating and tonic effects of adaptogens are well documented in numerous publications and reviewed in *Phytomedicine* [77]. In contrast to conventional stimulants, such as sympathomimetics (e.g., ephedrine, fenfluramine, phentermine, prolintane) and general tonics, the adaptogens don’t possess addiction, tolerance and abuse potentials, they don’t impair mental function and lead to psychotic symptoms in long term use, Table 4. Their clinical and pharmacological effects are due to a different mode of action. Their stimulating effect is more pronounced against a background of fatigue and stress.

**Table 4:** The differences in properties between adaptogens and other stimulants, adapted from [77,78,113,136].

Characteristic		Stimulants	Adaptogens
1.	Recovery process after exhaustive physical load	Low	High
2.	Energy depletion	Yes	No
3.	Performance in stress	–	Increased
4.	Survival in stress	–	Increased
5.	Quality of arousal	Poor	Good
6.	Addiction potential	Yes	No
7.	Side effects	Yes	Rare
8.	DNA/RNA and proteins synthesis	Decreased	Increased

## 4. Clinical Studies

The most important characteristics of adaptogens, such as stress-protection and a stimulatory effect are common to all adaptogens. However, the effects may differ under various circumstances (Table 1 and Table 2) as has been documented in a number of clinical studies (Table 5) and reviews [14,23,26,28,31,32,36,37,77,78,112,113,137,138,139,140].

The majority of the reviews focused on narrative observations of clinical studies and only a handful conducted a systematic assessment and quality scoring of the level of evidence as recommended by the Natural Standards and the EMEA [23,77].

One such review [31] focused on over 35 clinical trials on *Eleutherococcus* *senticosus* in healthy human subjects (ca. 6,000 subjects aged from 19 to 72), which were performed in normal and stressful conditions (e.g., high temperature environment, forced work periods, loud noise conditions, motion sickness, varying degrees of deafness, heavy physical burden, hypertension, mountain rescuers under forced conditions, athletes, deep sea divers, intense mental work and physical work, factory workers under extreme working condition). Farnsworth *et* *al*. observed that there was an improvement of the physical and mental work capacities in all cases. In addition, over 35 studies have focused on the effect of *Eleutherococcus* *senticosus* on more than 2,200 sick patients [31]. The studies included patients with atherosclerosis, acute pyelonephritis, diabetes, hypertension, trauma, neuroses, rheumatic heart disease, chronic bronchitis, insomnia, cancer and several other ailments. In most cases, a moderate improvement relative to the initial conditions was observed. The extracts were well tolerated and no side effects were observed [31,32].

However, the most convincing evidences of the efficacy of adaptogens were found in studies related to its neuro-protective effects, effects on cognitive functions and mental performance in fatigue, and on its efficacy in asthenia and depression [23]. The evidence points to adaptogens may be beneficial on neurodegenerative disorders [54,83,84,89,135,141].

### 4.1. Adaptogens in Fatigue, Effect on Cognitive Functions

In total, more than 30 publications on the clinical efficacy of various *Rhodiola* *rosea* preparations can be found in the PubMed database. The majority of these studies are of varying methodological rigor and concern cognitive functions and mental performance under fatigue, Table 5.

The clinical trials using *Schisandra* *chinensis* (13 studies) and *Eleutherococcus* *senticosus* (11 studies) on mental performance in humans have been the subject of a recent review [23]. A systematic review showed that adaptogens have a significant, beneficial and specific effect on stress-induced symptoms under fatigue [23]. It was seen that *Rhodiola* *rosea*, in particular, significantly reduced symptoms of fatigue and improved attention after four weeks of repeated administration [122]. Furthermore, it was suggested that the inhibitory effect of *Rhodiola* *rosea* on the increased basal level of salivary cortisol results in an improvement of cognitive function. This is in line with other studies demonstrating that optimal corticosteroid levels are a requirement for efficient cognitive function. It has been shown that significant changes (upregulation or downregulation) of circulating levels of corticosteroids are associated with cognitive impairment [168]. Moreover, studies on healthy volunteers receiving single and repeated doses of SHR-5 extract (*Rhodiola* *rosea*) have demonstrated an anti-fatigue effect and improvement in cognitive functions during fatigue and in stressful conditions [143,144,145]. Thus, one may conclude that repeated administration of *R.* *rosea* extract (SHR-5) exerts an anti-fatigue effect on healthy subjects and burnout patients expressing fatigue syndrome. This in turn, increases the patient’s mental performance and ability to concentrate.

**Table 5:** Results of clinical studies on humans involving effects of plant adaptogens on physical and mental performance related to fatigue.

Plant name Intervention/ control/ Daily dose/ Duration	Study design^a^/ Total subjects	Primary endpoint and main results^b^	Quality level ofevidence*	Jadad score [183]*	Ref.
*Rhodiola rosea* Extract SHR-5 (288 mg twice daily)/placebo for 4 weeks *Rhodiola rosea*Extract SHR-5 (170 mg once daily)/placebo for 2 weeks *Rhodiola rosea* Extract SHR-5 (50 mg twice daily)/placebo for 20 days *Rhodiola rosea* Extract SHR-5 (single dose of 370 mg or 555 mg) /placebo *Rhodiola rosea* Extract SHR-5 (170 mg or 340 mg twice daily)/placebo for 6 weeks	R, PC, DB 2 parallel groups 60 volunteers with stress-induced fatigue, (30/30) [20–55 years]	Symptoms of fatigue, attention, depression, QOL, salivary cortisol. Symptoms of fatigue, attention and salivary cortisol significantly improved compared with control	Ib	5	[122]
	R, PC, CO, DB 2 parallel groups 56 healthy subjects (?/?)^c^ [24–35 years]	Mental fatigue, perceptive and cognitive functions such as associative thinking, short-term memory, calculation and ability of concentration, and speed of audio-visual perception Statistically significant improvement in the treatment group (SHR-5) during the first 2 week period	Ib	4	[143]
	R, PC, DB 2 parallel groups 40 healthy subjects (20/20) [17–19 years]	Mental fatigue, physical performance, general well-being Significant improvement in physical fitness, mental fatigue and neuromotor tests compared with control (p<0.01). General well-being was also significantly (p<0.05) better in the verum group. No significance was seen in the correction of text tests or a neuromuscular tapping test	Ib	3	[144]
	R, PC, DB 3 parallel treatments	Capacity for mental work Significant difference in anti-fatigue effects in SHR-5	Ib	3	[145]
	groups 161 healthy subjects, (41/20/40 treated + 20 untreated) [19–21 years]	groups compared with control (p<0.001), whilst no significant difference between the two dosage groups was observed.			
	R, PC, DB 3 parallel treatment groups 91 patients with mild and moderate depression (31/30/30) [18–70 years]	Depression in total HAMD and BDI scores Significant differences in HAMD and BDI scores and scores reflecting levels of insomnia, emotional instability, somatisation and self–esteem in SHR–5 groups compared to placebo (p<0.001)	Ib	5	[134]
*Eleutherococcus senticosus* Extract (2mg eleutherosides B and E equivalent to 2–4 g of herbal substance daily)/placebo for 2 months and 2 months of follow-up	R, PC, DB 2 parallel treatment groups 96 patients with chronic fatigue syndrome (49/47) [21–65 years]	RVI measurement of fatigue reduced Significant (p<0.05) improvement in RVI compared with control after 2 months treatment in the subgroups of patients with moderate fatigue at baseline (RVI value 8–12) and a history of fatigue <5 years. However, no significant difference was observed after 4 months treatment	Ib	5	[142]
Chisan-fixed combination of Rhodiola-Schisandra-Eleutherococcus	R, PC, DB 2 parallel groups 60 patients suffering from acute non-specific pneumonia (30/30) [18–65 years]	Duration of antibiotic therapy associated with the clinical manifestations of the acute phase of the disease, mental performance and self-evaluation by WHO-QOL brief questionnaires Adjuvant therapy with ADAPT-232 had a positive effect on the recovery of patients by decreasing the duration of the acute phase of the illness for 2 days, by increasing mental performance of patients in the rehabilitation period, and by improving their QOL	Ib	4	[146]
ADAPT-232 capsules [fixed combination of standardised extracts of *Rhodiola rosea* (salidroside 3 mg), *E. senticosus* (syringin 3 mg), *S. chinensis* (schizandrin 4 mg)] 3 capsules *acute*	PC, CO, DB 60 PC, CO, DB 5 cosmonauts	Compared with placebo, ADAPT-232 significantly increased short-term memory, speed and reliability in the understanding of information, and precision and accuracy in the ability to reproduce the information in repeated highly sophisticated computer-based tests (Monotonic 2). ADAPT-232 was most effective against a background of pronounced fatigue induced by monotonous night work. The effect was most marked in complicated tests and under extreme conditions.ADAPT-232 significantly decreased the number of mistakes (*cf.* with placebo) in complicated psychometric tests 4 h after administration and increased working capacity 1.5 and 4 h after administration to Russian cosmonauts during their training in prolonged isolation (90 days) under conditions of long-term, monotonous work. No significant effects were observed in non-complicated tests.	IIa IIa	1	[147] [148]
*Eleutherococcus senticosus* extract 0.25, 0.5, 1.0, 2.0, 4.0 and 8.0 mL *acute*	PC, CO, OL 357 Sailors	*E. senticosus* improved mental performance in correction test; increased activity of the adrenal cortex, the activity of the sympathetic adrenomedullar system, the intensity of metabolic processes, and the intensity of red-ox processes. In stress conditions *E. senticosus* decreased adrenal cortex activity and sympathetic nervous system; increased the tonus of the parasympathetic nervous system; moderately intensified excitation of the CNS and of energy metabolism; improved endurance to hypoxia.	IIa	1	[149]
*Schisandra chinensis* extract 0.25, 0.5, 1.0, 2.0, 4.0 and 8.0 mLacute		*S. chinensis* stimulated the activity of the CNS at night; increased tonus of the sympathetic part of the autonomic nervous system (having no effect on the parasympathetic part) after night duty; activated the adrenal cortex activity; increased the activity of the cardiovascular and respiratory systems; intensified oxidation-reduction and metabolic processes; improved working ability parameters; reduced parameters of non-specific resistance the organism.			
*Rhodiola rosea* (extract) 10 drops or 3 x 10 drops/day *Rhaponticum cartamoides* (extract) 40 drops or 3 x 10 drops/day. Acute and 10 days	PC, SB 80 healthy students (control group) and 70 patients with neurosis	Single and repeated administration of adaptogens improved functional state of the CNS in patients with neurosis as characterised by normalisation of the speed and power of neural processes in Ivanov-Smolenski’s verbal test with speech-supported locomotor-conditioned reflex measurement. The memory improved and attention became more stable.	IIa	1	[150]
Rhodosin (R. rosea extract, 100 mg/ 20 days	PC, DB 60	Significant improvement in physical fitness, mental fatigue and neuromotor tests compared with control (p<0.01): general well-being was also significantly (p<0.05) better in the verum group. No significance was seen in the correction of text tests or a neuromuscular tapping test.	IIa	1	[151]
Rodelim tablets (fixed combination of the extracts of *R. rosea,* *E. senticosus* and *S. chinensis*) 100 mg/acute	PC, DB 60	Rodelim improved mental working capacity in computer and correction tests against a background of fatigue.	IIa	1	[152]
*R. rosea* (tincture 40% ethanol) 5-10 drops/acute	PC/85	Improved mental performance, reduced the number of errors in Anfimov’s correction test: the stimulating effect lasted 4 h or more	IIb	1	[153]
*Rhodiola rosea* (40% ethanol tincture, 20 drops) *E. senticosus* (40% ethanol tincture, 20 drops) Extract of *R. rosea* rhizome, 0.3 g/ acute	PC/254	Improved mental performance; reduced the number of errors in Anfimov’s correction test; increased the accuracy, working capacity and speed of information perception. Stimulating effect lasted 4 h or more	IIa	1	[154]
Tyrosol (1, 5, 10 and 20 mg), *R. rosea* extract, 5 drops	? / 82 patients	Improved mental performance, reduced the number of errors in Anfimov’s correction test.	III	0	[155]
Salidroside, 2.5 mg/ acute	PC, SB/ 46	Improved mental performance; reduced the number of errors in Anfimov’s correction test; stimulating effect lasting 4 h or more.	IIa	1	[39]
*E. senticosus* (dry extract), 120 mg/ Acute and 3 weeks	PC, DB/40	Improved mental performance determined by a letter correction test	IIa	1	[156]
*E. senticosus* (tincture) *P. ginseng* tincture/ 2 ml/ acute	PC, SB,CO/ 13	Decreased errors in data sent by radio operators 1 h after drug uptake. Stimulating effect of *E. senticosus* was stronger and more stable than the effect of Ginseng, which was insignificant.	IIa	1	[157]
Eleutherococcus (extract 1250 mg) Ginkgo (extract, 28 mg flavonoids, 7 mg gingkolides) 3 months	PC, CO /24	Selective memory significantly improved compared with placebo (p<0.02).	IIa	1	[158]
*S. chinensis* (tincture) *E. senticosus* (tincture) *Rhaponticum cartamoides* (tincture) *Oplopanax elatus* (tincture), 0.5, 1.0 and 2.0 mL, acute	PC, CO, OL 1327 healthy subjects	Eleutherococcus and Rhaponticum treatment significantly increased precision in tremometric test compared with placebo. Significant differences compared with placebo were observed with various doses of all tested preparations in psychometric tests including assessment of attention and memory functions.	IIa	1	[159]
*S. chinensis* (tincture) *E. senticosus* (tincture) *Rhaponticum cartamoides* (tincture) *Oplopanax elatus* (tincture, 0.5, 1.0 and 2.0 mL), 10 days	PC, CO 665 healthy subjects	Pilots were tested before a flight and again 5 –15 min later, and finally 1 and 3 h after landing Significant differences compared with placebo were observed for all tested preparations in tests including assessment of precision, dynamic tremometry, sensomotor response, and attention and memory functions	IIa	1	[160]
*S. chinensis* (tincture, 2 ml) *E. senticosus* (tincture, 2 ml) Coffee, 200 ml Black tea, 200 ml (control) Acute and repeated	OL, PC 200 sailors	*S. chinensis* showed a tonic effect for 4–7 h in those on night duties. *E. senticosus* was inactive Repeated uptake (for more than 2 weeks) of coffee and *S. chinensis* produced similar negative effects (insomnia, excitability, etc.).	IIb	0	[161]
*S. chinensis* (seed powder, 2g) *Panax ginseng* (tincture, 2 ml), acute	CO, PC, SB, 122 students	Improved attention in text correction 2 h after drug administration. Both extracts increased quality and quantity of mental work performed.	IIa	1	[162]
*S. chinensis* (seed tincture, 30 ml) *P. ginseng* (3% tincture, 30 ml) Phenamine(0.02 g) Glucose (0.5 g), acute	CO, PC /20	*P. ginseng* and *S. chinensis* improved accuracy in the work of telegraph operators at exhaustion compared with control group (glucose) and those given phenamine.	IIa	1	[51]
*S. chinensis* (seed – 3 g, extract and fractions, tablets and capsules, 0.036–0.168 g), acute	OL,CO/20	Improved accuracy in error correction test. Most active stimulating effect was revealed by a crystalline substance identified as the lignan schizandrin.	IIb	0	[50, 51]
Schizandrin (0.02, 0.01, 0.005 g), Phenamine (0.2 g) Glucose (0.5g) acute	PC/23	Schizandrin improved accuracy in the work of telegraph operators at exhaustion compared with control group (glucose) and those given phenamine.	IIb	1	[29,51]
*S. chinensis (*seed powder, 0.25 and 0.5 g), *10 days*	OL / 36	Beneficial effects on symptoms of astheno-depressive syndrome	IIIa	0	[163]
*S. chinensis* (fruit and seed tincture, 1:5, 90% ethanol, 1 ml), 16–40 days	OL/ 40	Stimulating effect-improve mood, increases physical and psychological vivacity, relief of tiredness and fatigue in asthenia and depressions.	III	0	[164]
*S. chinensis* (tincture, decoction and tablets), 2–10 weeks	OL/ 2000	Stimulating and tonic effct.	III	0	[165]
*S. chinensis* (tincture, decoction and tablets), 2–10 weeks	OL/ 250	Effective in the treatment of general asthenia, exhaustion and reduced physical and mental performance in group of patients with nervous disorders where an increase in general well-being and working capacity, as well as a decrease in sleepiness and exhaustion, were observed.	III	0	[166]
*S. chinensis* (tincture 95% ) 45–120 drops *S. chinensis* (tablets, 0.25 g, 0.5 g), *10 days*	OL, observational study/ 30	Stimulating effect in astheno-depressive syndrome with relief of somnolence, limpness, tiredness, fatigue	III	0	[167]

**^a^** R – Randomized, PC – placebo– controlled; DB – double– blind; SB – single blind, NC – not controlled; CO – crossover, UC – uncontrolled, OL – open label trial; **^b^** According to WHO, FDA and EMEA: Ia – meta– analyses of randomised and controlled studies; Ib – evidence from at least one randomised study with control ; IIa – evidence from at least one well– performed study with control group; IIb – evidence from at least one well– performed quasi– experimental study; III – evidence from well– performed non– experimental descriptive studies as well as comparative studies, correlation studies and case– studies; and IV – evidence from expert committee reports or appraisals and/or clinical experiences by prominent authorities; ^c^ ? – data not listed or unavailable.* –max 5.

### 4.2. Adaptogens in Asthenia and Psychiatric Disorders

It should be mentioned, that the majority of clinical studies cited here are the most questionable and poorly documented as standardized psychological measures were not used in the earlier studies. Indeed, some of them did not use randomization, or blinding of subjects. However, the main problem in assessment of these studies is that the Soviet diagnostic criteria were different from commonly used criteria in the rest of the world [169,170,171]. The diagnostic criteria used in the USSR prior to 1990 for schizophrenia was particularly idiosyncratic, overused, and misapplied to other conditions [169,170,171]. For example, some patients who would be diagnosed as having psychotic depression, schizotypal disorder, schizoaffective disorder, or bipolar disorder by a non-Soviet psychiatrist would have been diagnosed as schizophrenic by a Soviet psychiatrists [166,167,168]. Indeed, the term “sluggish-schizophrenia” was particularly misused [172]. The diagnoses of asthenia and neuroasthenia include a very heterogeneous group of patients with mixed psychological and physical disorders, making the studies more difficult to interpret.

Nevertheless, in spite of numerous shortcomings, which reduced the quality of evidences obtained in the early clinical studies in Russia, these scientific evidences provide important information about efficacy and safety of adaptogens in the treatment of psychiatric disorders, table 6. Some of these publications are discussed below.

**Table 6:** Summary of clinical trials of *Rhodiola* *rosea* and *Schisandra* preparations in psychiatric disorders.

Pathophysiological condition: Pharmacological activity or effect recorded	Plant name	Reference	Study design	Quality in Jadad’s score	Number of patients
**Fatigue syndrome:**	*R.rosea*	Olsson, 2009	R,PC,DB	5	60
**Adults physical and cognitive deficiency**	*R.rosea*	Fintelman, 2007	OL	0	120
**Depression: **	*R.rosea*	Darbinyan, 2007	R,PC,DB	5	91
	*R.rosea* **	Brychenko, 1987**	OL, C**	0	78/56**
	*S.chinensis*	Staritsina, 1946	OL, UC	0	37
**Astheno-depressive syndrom (stress-induced mild depression):**	*R.rosea*	Krasik, 1970*	OL, UC	0	128
	*R.rosea*	Krasik, 1970*	OL, UC	0	135/27
	*R.rosea*	Mikhailova, 1983	OL	0	58
	*R.rosea*	Mesheryakova, 1975	OL	0	25
	*S.chinensis*	Zakharov, 1956	OL, UC	0	13
	*S.chinensis*	Leman, 1952	OL, UC	0	40
	S.chinensis	Rossijskij, 1952	OL, Uc	0	260
**Neurosis (sress-induced depression )**	*R.rosea*	Saratikov, 1965*	OL	0	65
	*R.rosea*	Kaliko, 1966	PC, SB	1	70/80^$^
	*S.chinensis*	Sudakov, 1986	OL, UC	0	386*
**Anxiety:**	*R.rosea*	Bystritsky, 2008	OL	0	10
**Schizophrenia**	*S.chinensis*	Romas, 1958, 1962	OL	0	79/41*
	*S.chinensis*	Zakharova, 1948*	OL, C	0	30/20*
	*S.chinensis*	Lastovetskiy,	OL	0	48

**^a^** R – Randomized, PC – placebo– controlled; DB – double– blind; SB – single blind, NC – not controlled; CO – crossover, UC – uncontrolled, OL – open label trial; ^c^ ? – data not listed or unavailable.* – mixed patients population, sick/heatly subjects. ** – adjuvant therapy with antidepressants, control group – tricyclic antidepressants. $ – sick/heatly subjects.

#### 4.2.1. *Rhodiola Rosea*

In 1969, the Pharmacological Committee of the Ministry of Health of the USSR recommended the medicinal use of *Rhodiola* *rosea* extract in patients suffering from illnesses, such as asthenia syndrome, neuroses, vascular dystonia, hypotension, and schizophrenia (remissions of asthenic type). The advised dosage of the extract was 5–25 drops (in a quarter of a glass of water) three times daily and should be taken 15–30 minutes before meals. Although, the duration of the therapy was individually determined and varied from 10 days to 4 months [173,174].

The symptomatic characteristics of asthenic syndrome is general weakness, reduced working capacity, low memory, irritability, headaches, insomnia, and poor appetite. These symptoms are frequently observed after intensive work, which requires high levels of mental exertion. Indeed, the *Rhodiola* *rosea* extract was reported to be a valuable medicine in essentially healthy people placed under heavy physical or mental workloads [175,176]. Furthermore, to take advantage of the prophylactic measures of *Rhodiola* *rosea* extract it has been advised that doses should be taken in the morning, or in the morning and the afternoon, for at least a few days prior to a period of intensive mental work for a during 2–3 weeks. In cases of hard mental work, it has been recommended that duration of *Rhodiola* *rosea* therapy last for the duration of the period. It has been reported that therapy with *Rhodiola* *rosea* extract prevented exhaustion and fatigue during work requiring long intensive mental activity [175,176].

The positive therapeutic effect of *Rhodiola* *rosea* extract was also observed in an open label, uncontrolled study in 128 patients with pronounced states of fatigue of different genesis [175]. For example, 128 patients between 17 and 55 years of age (male/female: 75/53) were treated with an *R.* *rosea* extract and as a result the clinical symptoms of fatigue were considerably improved or disappeared completely. Their diagnosis of fatigue was confirmed by psychological examinations and increased mental work capacity. Indeed, there are also therapeutic effects of *Rhodiola* *rosea* extract in exhaustion due to psychogenic and somatogenic origin (82%) or in patients convalescing from somatic and infectious diseases (80%) [175]. For example, patients suffering from post-influenza fatigue showed improved mental and physical working capacity already on the third day after intake of *Rhodiola* *rosea* extract. Thus, these patients improved in focusing their attention, and headaches vanished. Furthermore, *Rhodiola* *rosea* extract reduced and eliminated general weakness and fatigue in cases of traumatic cerebrastenia injury and also significantly facilitated the normalisation of the autonomic functions in this group of patients [175].

That *Rhodiola* *rosea* extract has a pronounced therapeutic effect on neuroses (exhaustings depression) was reported in an open label study on 65 poorly characterized mixed patient population [177] suffering from insomnia, high irritability and various somatic disorders. The state of the higher nervous activity was tested in these patients with the help of a verbal test and the conditional motor reflexes were tested with verbal reinforcement, in conjunction with routine clinical examinations. The results of the verbal tests showed that the latent period of verbal reactions in most of the subjects was long-up to 1.8–6.0 s (a normal period is 1.5 s). Indeed, results of the motor-verbal test prior to treatment showed that two thirds of the patients performed poorly in mental work situations. However, after a course of therapy with *Rhodiola* *rosea* extract (10 drops; 3 times daily for 10 days) the excitation and inhibitory processes intensified and mobility normalized. In fact, the conditional motor reflexes also changed. It was seen that their value and stability increased and that the latent period reduced, whilst concentration improved and the interaction of both signal systems were normalized. Furthermore, the extract therapy reduced the latent time of verbal reactions and improved attention and memory.

Indeed, the authors observed a clinical improvement, such as disappearance of irritability and unpleasant sensations in the heart region and improvement of sleep and appetite, in these patients. Similar clinical results were obtained in another study with 177 patients suffering from vascular hypotension. The authors observed that a course of therapy with *Rhodiola* *rosea* extract stabilized, completed or partially normalized 92% of the hypotensive patients. Thus, the patients felt better, headaches disappeared, sleep became normal and they recovered their working capacity.

Several clinical studies of the dynamics of asthenic symptoms have showed that *Rhodiola* *rosea* extract was able to diminish or eliminate depressive symptoms. For example, Mikhailova reported that an open label clinical trial of *Rhodiola* extract (15 drops three times daily for a month or up to four months in some cases) in 58 patients with stress induced depression (asthenia of exogenous organic genesis), showed a remarkable symptomatic improvenment was observed [178]. Their general weakness or feeling of being worn out in the morning with a high fatigability, and hypersomnia during the day (without disturbance of the following night’s sleep) either disappeared or became considerably reduced. In addition, no side effects were observed except in one patient who showed sleep disturbance. In this case, the dose was reduced to 6 drops twice a day and the adverse symptoms immediately disappeared. Indeed, most patients (39/58) suffering from pre-and intra somnia (*i.e.,* difficulties in falling asleep, waking up during the night) noted sleep improvements. However, it should be noted that sleep normalisation and reduction of other asthenic symptoms depended on the intensity of these disorders [178].

Furthermore, it was seen that patients in depressive states of different genesis normally treated with tricyclic anti-depressants reduced the time spent in hospital whilst using them in combination with *Rhodiola* *rosea* [179]. The authors observed adjuvant therapy with *R.* *rosea* increased general activity, intellectual and physical productivity of patients, and decreases side effects induced by the tricyclic anti-depressants.

In was reported that *Rhodiola* *rosea* extract was successfully used in the alleviation of side effects after psychotropic therapy in schizophrenic patients [176]. In this short communication is mentioned that 31 patients suffering from pronounced clinical symptoms of extrapyramidal syndrome, which resulted from therapy by neuroleptics, received a high dose of *Rhodiola* *rosea* extract (25–40 drops; 2–3 times daily) for a duration of 1–1.5 months. In addition, 19 of these patients received a combination of a high dose of *Rhodiola* *rosea* extract and cholinolytic Romparkin (Trihexylphenidyl), as this preparation alone failed to eliminate or soothe the clinical symptoms of the side effects in this group of patients. The results of this study showed that *Rhodiola* *rosea* extract had the most pronounced therapeutic effect on symptoms of Parkinsonism and asthenia.

In conclusion, the introduction of *Rhodiola* *rosea* extract in the USSR was based on the following findings:

(i) as stimulants for essentially healthy people in a state of fatigue and for patients with asthenic states during the rehabilitation period following somatic or infectious diseases.
(ii) for essentially healthy people *Rhodiola* *rosea* extract should be taken several days before the expected strain and during the whole period of increased mental exertion as a prophylaxis.
(iii) to recover mental and physical working capacity during and after long periods of intensive mental and physical work.
(iv) and sexual disorders related to impotence in males.
(v) in psychiatric practice as an adjuvant to counteract side effects of psychopharmacological therapy [28].

Furthermore, side effects of therapy with *R.* *rosea* extract are relatively rare. Although, some cases of individual sensitivity to the preparation have been observed, such as excitement, irritability, insomnia, and headache.

#### 4.2.2. Schisandra chinensis

*Schisandra* *chinensis* extracts (e.g., tincture, decoction and tablets) have been reported to be effective in the treatment of general asthenia, exhaustion and reduced physical and mental performance in open label studies on a population of 2,000 poorly characterized mixed patients. It was reported that the extracts showed improvement after 2–10 weeks of therapy [165,166]. Furthermore, the treatment showed a particularly remarkable effect on a group of patients (n = 200) with nervous disorders where an increase in general well-being and working capacity, as well as a decrease in sleepiness and exhaustion [165,166]. Another study [180], which was based on 13 patients, concluded that two administrations of *S.* *chinensis* extract per day was optimal and that prolonged treatment may cause negative effects. The author pointed out that the duration of treatment should be decided on an individual patient basis. Taken together, the results of the treatment showed considerably improved results in short term patients, in comparison to chronic patients [180].

A comparative clinical pilot study of *S.* *chinensis* seeds (tincture) in neurasthenic patients (n = 95) showed that there was a high efficiency present in the therapy (dosage: 15 drops during 25–28 days). The results showed that general weakness, poor sleep and appetite, high irritability and headaches disappeared almost completely in the treatment group, whilst 55% of the control group complained of these symptoms. In addition, the muscular force of the hands increased around 2.5 times in the treatment group, in comparison to the control group. It was also seen that the gain of vital lung capacity reached 19% in the treatment group, in comparison to 3% in the control group [181].

Several studies have reported a positive therapeutic effect of *S.* *chinensis* preparations on asternic-and astheno-depressive states (particularly in stress induced depressions) snd schizophrenia [184,185,186,187,188,189,190,191,192]. For example, Leman observed a stimulatory effect of a *S.* *chinensis* preparation on the CNS after 16–40 days of treatment in 40 patients with asthenia and depressions of psychogenic or somatic origin [164]. There was also an improvement in dark vision found in almost all patients. Twenty two patients felt pleasantly warm, became more energetic and physically active, whilst hunger and fatigue disappeared. In addition, their mood improved and night sleep was normalised. The authors concluded that a therapy with *Schisandra* *chinensis* can be indicated in asthenia and depression of psychogenic etiology, so called “exogenous” (stress induced) depression, or states related to excessive fatigue, somatic and nervous exhaustion. However, they also reported that the therapy can only relieve symptoms in “endogenous” depression (organic etiology), asthenia, narcoleptic and amnestic syndromes. An advantage of treatment with *S.* *chinensis* is the absence of serious side effects. Therefore, the tolerance to *S.* *chinensis* extract is several times higher than the tolerance to caffeine or phenamine, and the effect is not reduced following a long treatment period.

Another study reported total recovery of psychosis following treatment with *S.* *chinensis* extract [182]. The authors treated 36 patients (19 with schizophrenia; six with reactive psychosis; four with alcoholic psychosis; three with depression; and four with psychopathy) with ADS three times daily with *S.* *chinensis* preparations. All in all, total recovery was observed in patients suffering from psychosis, while no effects were seen in patients with diagnosed psychopathy.

Furthermore, a tincture of *S.* *chinensis* was used in treatment of schizophrenia (79 patients) and chronic alcoholism (118 patients and 41 healthy subjects) in an open label observational trials) [184]. The author measured pupillary and vascular (alterations of palm vessel volume) tentative reactions, and refectory reactions in order to evaluate the effects on the CNS. These reactions were comparatively suppressed in schizophrenia and chronic alcoholism. However, it was shown that treatment with *S.* *chinensis* tincture normalised the reactions in the patients under study and activated reactions in healthy subjects. As a result of the treatment, patients became calm, sociable, and active, free of emotional tension and anxiety, and willing to work. Indeed, they showed excellent well-being, which was associated with a good mood. The author reported an optimal dose of 15–25 drops in simple and hallucinogenic-paranoidal schizophrenia, 5–15 drops in paranoidal schizophrenia, and 5 drops in catatonic schizophrenia [184].

Furthermore, two open, uncotroled studies [184,185] reported a somewhat unexpected finding in that there was a stimulatory effect in schizophrenic patients and chronic alcoholics. This was due to the ability of the *S.* *chinensis* extract to increase the reactivity to insulin, sulphadiazine and apomorphine. Thus, administration of a *S.* *chinensis* preparation, which was used in conjunction with apomorphine, frequently eliminated or decreased addiction to apomorphine. The authors noted development of a stable conditional emetic reflex in most of the chronic alcoholics at the end of the treatment [184,185,186]. It was also found that *S.* *chinensis* treatment increased associative processes in the subjects and improved the quality of associations, as demonstrated by the increased number of higher verbal responses and a decrease in the number of lower responses [187].

It was also reported that *Schisandra* *chinensis* extract had a positive effect in an open, uncontroled study on 386 patients with nervous and/or mental disorders of borderline exogenous-organic genesis (stress induced mild depression) [188]. These positive therapeutic effects were the result of a stimulating action and a prevention, or reduction, of development of side effects associated with treatment with tranquilizers and anti-depressants. Furthermore, the combination of *Schisandra* *chinensis* extract with nootropic preparations (e.g., nootropil, pyracetam) was found to be effective in patients with pronounced mild cognitive (mnestico-intellectual) disorders. Although, in patients with agitative dementia the *Schisandra* *chinensis* therapy was less effective [188]. Another observation, which could be of practical future importance, is that a combination of *S.* *chinensis* therapy with tranquilizers or anti-depressants (e.g*.*, amitriptyline, relanium) may be very effective in the elimination of the undesirable effects of these drugs. For example, the development of side effects (e.g., headaches, dizziness, flaccidity, dryness in the mouth, and urination disorders) were observed in 23 out of 39 patients (53.5%) with neuro-mental disorders of exogenous-organic genesis, which when treated with increased doses of amitriptyline (from 50 to 75 mg). However, patients treated with *S.* *chinensis* experienced a similar effect in only 4 out of 172 patients (1.9%). Thus, a combined administration of tranquilisers and adaptogens permitted the usage of optimal doses of these drugs in 96% of patients, whilst it was only possible in 16% of control patients (p < 0.001). It has been concluded that:

(i) *S.* *chinensis* extract can be used in psychiatric practice as a symptomatic agent against astheno-depressive states independent of the nature of the disease.
(ii) preparations of *S.* *chinensis* decrease fatigue, improve the general mood and appetite, and can be recommended as a tonic for healthy people in a state of fatigue. No negative effects were observed on the somatic state of patients.
(iii) S. *chinensis* extract can be used in the treatment of psychoses as a stimulant without harmful side effects.
(iv) the curative effect of *S.* *chinensis* preparations is pronounced in cases of asthenic and depressive syndromes.
(v) the combination of *S.* *chinensis* therapy with tranquilisers or anti-depressants eliminates the side effects of these drugs and allows them to be employed at optimal doses [188].

We may suggest, based on the reported studies, that *Schisandra* *chinensis* have a place in the treatment of schizophrenia in reduction of catatonia and negative symptoms, relief of fatigue, and amelioration of side effects from neurotropc and other sedating medications. However, the evidence for the use of *S.* *chinensis* in the treatment of schizophrenia is not scientifically rigorous. In our view, studies which claim that *S.* *chinensis* significantly improves symptoms of schizophrenia must be validated by accepted diagnostic criteria (ICD-10), rigorous methodology, and validated assessment tools before any conclusions may be drawn.

## 5. Conclusions and Perspectives of Implementation

Recent pharmacological studies of some adaptogens give a rationale to their effects at the molecular level. It has been shown that the beneficial stress-protective effect of adaptogens is related to the regulation of homeostasis via several mechanisms of action, which are associated with the hypothalamic-pituitary-adrenal (HPA) axis and the regulation of key mediators of the stress response, such as molecular chaperons (e.g., Hsp70), stress-activated c-Jun N-terminal protein kinase (JNK1), Forkhead box O (FoxO) transcription factor, cortisol and nitric oxide (NO). In summary, adaptogens may be regarded as a novel pharmacological category of anti-fatigue drugs that:

(i) induce increased attention and endurance in situations of decreased performance caused by fatigue and/or sensation of weakness.
(ii) reduce stress-induced impairments and disorders related to the function of stress (neuro-endocrine and immune) systems.

It was suggested that adaptogens have not only specific therapeutic effects in some stress-induced and stress-related disorders, but will also have an impact on the quality of life of patients when implemented as adjuvants in the standard therapy of many chronic diseases and pathological conditions (e.g., post-surgery recovery, asthenia, congestive heart failure, chronic obstructive pulmonary disease). It may be suggested that adaptogens have potential use in age related disorders, such as neurodegenerative diseases, and cardiovascular diseases. Thus, elderly people may be able to maintain their health status on a normal level, improve their quality of life and may increase longevity. However, further research may be needed to evaluate the efficacy of adaptogens as geriatrics and to elucidate molecular mechanisms of action of these complex herbal extracts and their active principles.
